# Exploring the Role of Hypoxia and HIF-1α in the Intersection of Type 2 Diabetes Mellitus and Endometrial Cancer

**DOI:** 10.3390/curroncol32020106

**Published:** 2025-02-13

**Authors:** Alagappan V. S. Geetha, Kannan Harithpriya, Kumar Ganesan, Kunka Mohanram Ramkumar

**Affiliations:** 1 Department of Biotechnology, School of Bioengineering, SRM Institute of Science and Technology, Kattankulathur 603203, India; ga1904@srmist.edu.in (A.V.S.G.); hk7829@srmist.edu.in (K.H.); 2 School of Chinese Medicine, Li Ka Shing Faculty of Medicine, The University of Hong Kong, Hong Kong, China; kumarg@hku.hk

**Keywords:** endometrial cancer, endometrial neoplasms, diabetes mellitus, HIF-1α, hypoxia

## Abstract

Diabetes and Cancer are the most complex chronic diseases, accounting for significant global mortality and morbidity. The association between Type 2 DM (T2DM) and endometrial cancer (EC) is multifaced, sharing numerous risk factors, including insulin resistance, obesity, hypoxia, and oxidative stress. Hypoxia plays a vital role in T2DM pathogenesis by altering the insulin level and pancreatic β-cell failure through an imbalance between antioxidant enzymes and cellular oxidative levels, while chronic inflammation contributes to EC malignancy. HIF-1α is a potent transcription factor involved in modulating cellular responses to hypoxia within the disease environment. Targeting the HIF-1α signaling cascade, a major metabolic regulator may contribute to advanced therapeutic advances. This review focuses on the association between T2DM and EC, especially focusing on hypoxia and HIF signaling pathways. These intersect with key pathways involved in T2DM and EC pathology, such as insulin signaling, PI3K/AKT, mTOR pathway, MUC1/HIF-1α pathway, and hormonal imbalance. Understanding this complex relationship paves the way for future researchers to develop HIF-1α-targeted therapies that could lead to novel combination therapies to treat these comorbid conditions.

## 1. Introduction

Diabetes and cancer are two complex, multi-factorial, heterogeneous chronic diseases that have a major impact on the quality and life expectancy [1]. Currently, diabetes ranks as the fifth most common cause of mortality worldwide, causing approximately 1.6 million deaths each year. A recent report predicts that the prevalence of diabetes will increase to 592 million by the year 2035 [2]. On the other hand, cancer is the second leading cause of mortality worldwide, with 17 million in 2018. Projections for 2040 present an even more concerning scenario, estimating 27.5 million new cases expected [3].

Extensive epidemiological research strongly suggests that diabetes is considered an independent risk factor associated with elevated rates of occurrence and mortality from diverse cancer types [4]. An umbrella review of observational studies by Stuttard and his team identified a strong association between genetically predisposed T2DM and six other cancer types, including colorectal, hepatocellular, gall bladder, breast, uterine, and pancreatic cancers [5]. In contrast, an inverse relationship was reported between T2DM and prostate cancer risk due to decreased testosterone levels observed in men with T2DM [6]. Additionally, no clear link between diabetes and the development of lung cancer has been recorded. The distribution of different lung cancer types (histology) appeared similar in participants with and without diabetes [7].

Among all the cancer types worldwide, EC (EC) ranks as the sixth most common malignancy diagnosed in women, with a mortality rate of about 4% in 2022, rising from 1997 [8,9]. The development of EC is characterized by various risk factors such as obesity, inadequate physical activity, nulliparous, menarche at an early age, estrogenic hormone replacement therapy (HRT), diabetes, hyperinsulinemia, and insulin resistance. Additionally, oxidative stress and chronic inflammation can influence the development of EC [10]. Although diabetes is recognized as a risk factor for EC, its effect on patient outcomes is still under discussion. A systemic review and meta-analysis carried out by McVicker and his research group in 2022 found a clear association between diabetes and poor patient outcomes, particularly a decrease in both cancer-specific survival and overall survival rates in women diagnosed with EC [11].

Analysis of existing epidemiological data shows that oral contraceptives containing both estrogen and progestin are associated with a reduced risk of EC, with the protective effect continuing significantly even after discontinuing use [12]. The impact of glucose-lowering drugs on cancer risk is still not clear, while associations among insulin, other medications, and comorbid risk have been proposed. The debate surrounding this connection requires further examination [13].

Oxidative stress (OS) occurs when there is an imbalance between the production of oxidants and antioxidant defense, potentially damaging biological systems [14]. OS is involved in the failure of pancreatic β-cells and affects insulin resistance by upregulating NF-κB activity, thus promoting the progression of T2DM. Research on animal models with T2DM showed that excessive intake of calories can lead to the accumulation of oxidative stress in adipose tissues, increasing the production of pro-inflammatory cytokines [15]. OS can also affect multiple pathways, promoting inflammation and endothelial dysfunction [16]. This inflammation can increase estrogen production and can favor the development of EC [17].

OS can also promote the development of EC by regulating various gene expressions. A study on endometrioid EC examined the mRNA and protein expression of genes associated with OS in EC and identified five genes involved in the cellular processes of ROS through mRNA microarray screening with 45 EC patients and 45 neoplastic participants. This included peroxiredoxin 2 (PRDX2), polycystin 2 (PKD2), aquaporin 1 (AQP1), superoxide dismutase 3 (SOD3), and Krueppel-like factor 2 (KLF2). PKD2 was found to be significantly overexpressed irrespective of cancer grade. SOD3, which plays a protective role against oxidative damage to proteins and lipids, showed decreased expression. PRDX2, AQP1, and KFL2 also showed reduced levels due to alterations in miRNA activities, indicating an association between miRNA and OS [18,19]. In a recent study, OS markers, such as ox-LDL, NO, and AGEs, were increased in patients with both T2DM and EC, which resulted in increased expression of OS stress marker 8-hydroxy-2′-deoxyguanosine, in association with decreased LCAT expression corresponding to T2DM and EC comorbidities [20].

Low oxygen levels, referred to as hypoxia, can increase the production of ROS and cause oxidative stress [21]. HIF-1 is a heterodimeric key transcription factor that is activated as a cellular response to hypoxia. Under hypoxic conditions, HIF-1α is stabilized by forming a dimer with the β subunit and is involved in various processes like angiogenesis, metabolic reprogramming, and cell proliferation, which can potentially promote the development of cancer [22]. Hence, measuring tumor hypoxia, oxidative stress markers, and antioxidant levels could be used as potential prognostic biomarkers in the future, particularly to treat EC [23].

Among all other cancer types, this review focuses on the complex association between T2DM and EC by discussing the epidemiology, pathogenesis, and possible molecular mechanisms. EC shares several characteristics with T2DM, including low oxygen conditions and the presence of adipose tissue, which is a major target in T2DM. Understanding the link between T2DM and EC, particularly the involvement of HIF-1α, might help future researchers predict the prognosis of targeting HIF-1α in individuals with comorbid conditions. This review also helps to identify potential biomarkers for the early detection of EC in diabetic patients and to predict outcomes in individuals with both disease conditions. A comprehensive review of scientific literature, including PubMed, Science Direct, EMBASE, Google Scholar, and Web of Science, was conducted to explore the role of hypoxia and HIF-1α in the intersection of type 2 diabetes mellitus and endometrial cancer.

## 2. Prevalence, Risk Factors, and Mutual Features of T2DM and EC

### 2.1. Etiology of Diabetes Mellitus

India, with 41 million cases, is considered the world’s “diabetes capital”, with one in five individuals diagnosed with diabetes globally being Indian [24]. Diabetes mellitus is characterized by hyperglycemia, which results from insulin resistance, inadequate insulin secretion, or dysregulated glucagon secretion [25]. Diabetes is associated with long-term impairment, malfunction, and failure of organs, especially the eyes, kidneys, nerves, heart, and blood vessels [26]. Earlier, diabetes mellitus is distinguished as an auto-immune (Type 1) and non-autoimmune (Type 2) disease. In contrast, other disorders are related to glucose regulation dysfunction, separately including gestational diabetes and monogenic diabetes [27].

T1DM arises from a malfunction of the body’s immune system, destroying insulin-producing pancreatic β-cells, resulting in a deficiency of insulin and hyperglycemia [28,29]. On the other hand, T2DM is characterized by insulin deficiency due to pancreatic β-cell malfunction and insulin resistance in key organs, resulting in a potential loss of up to 50% of β-cells by diagnosis [30]. T2DM is the leading form of diabetes, accounting for 90–95% of cases worldwide. Meanwhile, T1DM represents only 5–10% of reported cases [31]. Several factors influence the development of T2DM, including age, obesity, and an inactive lifestyle. However, the exact genetic mechanisms involved are complex and unclear. Unlike T1DM, T2DM originates in various tissues, with adipose tissue playing a key role. Changes in the insulin signaling pathway within these tissues lead to insulin resistance, a hallmark of T2DM. Understanding this pathway is crucial for developing effective treatments [32].

### 2.2. Etiology of EC

EC is the most common among all the other gynecological cancer types in highly developed countries, and its occurrence is on the rise among postmenopausal women. The most common symptom is abnormal uterine bleeding, which is present in approximately 90% of cases, often accompanied by vaginal discharge and pyometra (uterine infection) [33,34]. The exact origin of EC remains unclear, but numerous endogenous and exogenous risk factors have been linked to its occurrence, including genetic mutations disrupting important signaling pathways and activating oncogenes, elevated body weight and obesity, prolonged exposure to carcinogens, and changes in immune conditions that may facilitate cancer progression [35]. The primary risk factors associated with the onset of endometrioid endometrial carcinoma are obesity and chronic unopposed estrogen stimulation of the endometrium [36].

EC can be classified into different groups based on their histology and other characteristics [33,37,38,39]. EC was traditionally classified by Bokhman into Type I and Type II based on clinical and endocrine features. Type I EC is more common in obese women than Type II tumors, which are non-endometrioid histology [38]. Additionally, EC can be broadly classified based on histopathological characteristics and genetic profile. The subtypes include endometrioid, serous, or clear-cell adenocarcinoma. Genetic profiles include POLE (ultra mutated), MSI (hypermutated), copy-number low, and copy-number high [37].

### 2.3. Adipocytes: Common Ground for T2DM and EC

Adipose tissue is widely known as an energy storage reservoir and was recently recognized for its role as an endocrine organ that secretes cytokines, adipokines, and chemokines [40]. It is involved in metabolic processes, immune regulation, and the synthesis of biologically active compounds essential for cell development, angiogenesis, apoptosis, and carcinogenesis [41]. Adipose tissues secrete lipids and proteins responsible for endocrine activity and respond intensively to circulating hormones and metabolites, including growth hormones, insulin, cortisol, and lipids. It plays a critical role in maintaining glucose homeostasis in cells and organs. Adipocytes are the predominant cells in adipose tissue, which play a crucial role in maintaining the body’s energy homeostasis [42].

Obesity, a recognized risk factor for T2DM, is often accompanied by altered adipose tissue distribution [43]. The association of obesity with cancer development is linked to the excess adipocytes leading to tumor development in the breast, kidney, pancreas, colon-rectum, and endometrium. Hypertrophied adipocytes release significant levels of adipokines and growth factors during obesity. As these increase, the ability to function as endocrine cells exhibits an increase in expression for enzymes responsible for triglyceride synthesis. During periods of excess caloric intake, adipocytes trigger an inflammatory response, leading to adipocyte dysfunction. This provides energy for the growth and invasion of tumor cells [44]. Specifically in EC (EC), the interaction between adipocytes and EC cells involves high levels of estrogen, insulin, insulin-like growth factor-1 (IGF-1), adipokines like leptin, cytokines such as IL-6 and TNFα, and signaling pathways like VEGF-mTOR [45].

For instance, leptin binds to the leptin receptor present in the malignant tissues and promotes proliferation and survival, triggering a signaling cascade and initiating metastasis [46]. EC is predominantly linked with obesity, particularly through the conversion of androgens to estrogen. This conversion is mediated by the enzyme aromatase, which is expressed predominantly by adipocytes, preadipocytes, and mesenchymal stem cells in adipose tissues. High levels of estrogen play a significant role in endometrial proliferation in postmenopausal women [47]. The relative risk of developing EC in obese women is 6.3 times higher when compared to lean individuals. Additionally, metabolic syndrome and insulin resistance are linked with an increased risk of developing EC [48].

### 2.4. Metabolic Reprogramming

Glucose metabolism within EC (EC) cells involves a complex interplay of glycolysis and mitochondrial processes to meet energy requirements. Factors affecting glucose metabolism could potentially influence the onset and progression of EC [49]. Glycolysis serves as the primary source of energy in EC cells when oxidative phosphorylation (OXPHOS) is diminished or impaired. The metabolic pattern shifts between glycolysis and OXPHOS, depending on not only activated oncogenes within tumor cells but also by their surrounding microenvironment [50].

Elevated glycolysis levels have been observed in EC patients compared to healthy individuals, accompanied by increased expression of glycolysis-related enzymes such as GLUT, PKM2, LDH, HK, ENO1, PGI, and PK, as well as HIF-1α, a regulator of glycolysis, and lactic acid transporters, including MCT1 and MCT4 [51]. Molecular alterations associated with the diabetic phenotype, such as abnormalities in insulin signaling, which results in insensitivity to insulin and subsequent hyperinsulinemia, may ultimately favor tumor development through the regulation of HIF-1α, increased glucose uptake, and its utilization. The presence of tumor hypoxia is a significant contributor to alterations in metabolic enzymes and pathways in cancer. Hypoxia triggers the stabilization of HIF-1 and -2, where HIF-1 is known to upregulate various glycolytic enzymes.

The dominant mutation of succinate dehydrogenase (SDH) and recessive mutation of fumarate hydratase (FH) are enzymes involved in the TCA cycle that regulate HIF transcription factors and are often mutated in some cancers [52]. A recent study on EC tissues observed low expression levels of FH associated with increased tumor size and lymph node metastasis [53]. The high levels of fumarate and succinate resulting from these mutations might prevent the degradation of HIF protein by inhibiting the prolyl-hydroxylase enzyme. Thus, by disrupting the TCA cycle, tumor cells can stabilize HIF and enhance glycolysis, which helps in cancer progression [54].

**Figure 1:**
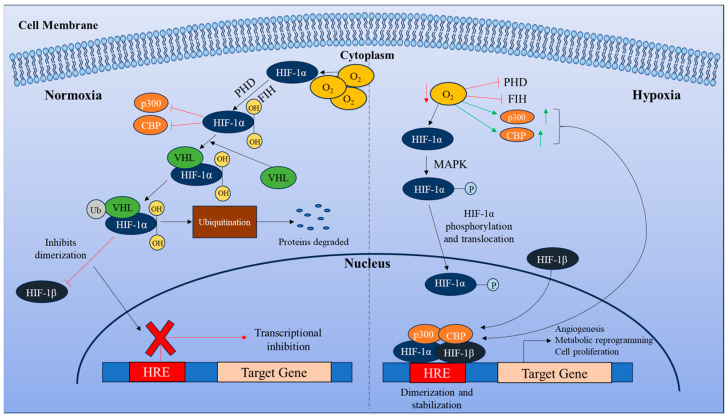
Differential regulation of HIF-1α signaling in normoxic and hypoxic conditions. Under normoxic conditions, HIF-1α undergoes hydroxylation by two oxygen-dependent enzymes, prolyl hydroxylases (PHDs) and factor-inhibiting HIF (FIH). PHDs facilitate the binding of von Hippel-Lindau (VHL), an E3 ubiquitin ligase, which ubiquitinates HIF-1α, leading to its proteasomal degradation. FIH inhibits the transcriptional activity of HIF-1α by preventing the recruitment of transcriptional coactivators, CREB-binding protein (CBP), and p300. In hypoxic conditions, HIF-1α is phosphorylated by mitogen-activated protein kinase (MAPK), which increases its stability. It is further translocated to the nucleus, where it dimerizes with HIF-1β to form a heterodimer. This complex, along with the recruitment of CBP/p300, enhances the transcription of HIF target genes. The HIF-1α/HIF-1β/CBP/p300 complex binds to the hypoxia-responsive element (HRE) of target genes, promoting angiogenesis, cell proliferation, and metabolic reprogramming.

Under hypoxic conditions, HIF-1α upregulates GLUT-1 and GLUT-3, essential for glycolysis and lactate production to maintain glucose homeostasis [55,56]. In T2DM, high glucose levels inhibit the stabilization of HIF-1α, impairing this adaptive response [57]. HIF-1α is overexpressed in EC cells and causes a metabolic shift known as the Warburg effect. This shift causes cancer cells to depend primarily on glycolysis for energy production, even in the presence of oxygen. This metabolic reprogramming promotes tumor growth and metastasis [58]. The expression and activity of HIF-1α can link the metabolic dysregulation in diabetes to cancer progression (Figure 1).

## 3. Signaling Cascades Involving HIF-1α Regulation

### 3.1. mTOR Pathway

mTOR is a conserved Ser/Thr protein kinase that exists as two multi-complexes, mTORC1 and mTORC2. These complexes can influence the protection or progression of OS through various signaling pathways. Metabolic homeostasis and the level of oxygen are mainly regulated by the expressions of mTOR and HIF-1α [59]. A recent study on oxidative stress showed that modified HIF-1α expression and mTOR gene expression would serve as a protective mechanism in patients diagnosed with T2DM [60]. Adding to it, a study conducted by Sam et al. highlighted that glucagon-like peptide-1 (GLP-1), an agonist and a class of medication used to treat T2DM, improves pancreatic islet viability by upregulating HIF-1α through cAMP-mediated induction of the mTOR pathway [61].

However, high levels of HIF-1α are associated with poor clinical outcomes of EC, making targeting HIF-1α an attractive treatment strategy. A recent study on women with severe endometriosis and infertility reported elevated expression of mTOR and HIF-1α in cancerous tissue from postmenopausal women with endometrial adenocarcinoma [46,62].

**Figure 2:**
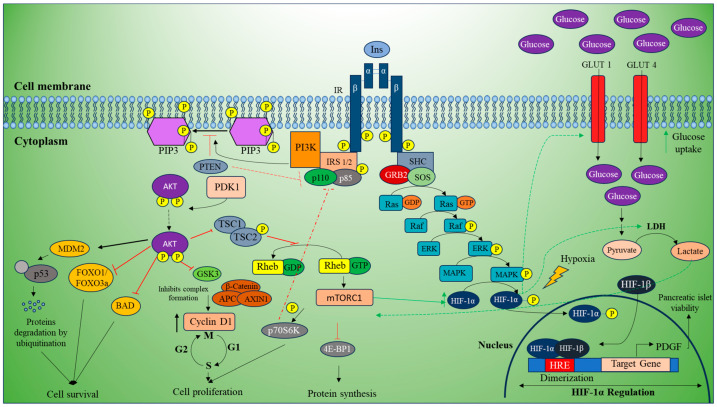
Insulin signaling and HIF-1α regulation in pancreatic cell survival and proliferation. Insulin binding to its receptor (IR) triggers autophosphorylation, recruiting IRS2, p110, and p85, which activate PI3K to convert PIP2 to PIP3. This recruits and activates Akt via mTORC2 and PDK1. Activated Akt promotes cell survival by inhibiting FOXO1/FOXO3a, BAD, p53, and GSK3 and supports mTOR signaling through Rheb. Concurrently, IR autophosphorylation recruits SHC, GRB2, and SOS, leading to Ras-GTP activation and MAPK-mediated phosphorylation of VHL.

On the other hand, autophosphorylation of the insulin receptor recruits the IRS2 gene, p110, and p85. This complex is phosphorylated and recruits PI3K to convert PIP2 to PIP3, which recruits Akt. Akt is double phosphorylated by mTORC2 complex and 3-phosphoinositide-dependent kinase 1 (PDK1). This activated Akt is observed to inhibit FOXO1/FOXO3a, BAD, and p53 by MDM-dependent proteasomal degradation by ubiquitination, which contributes to cell survival. Along with this, phosphorylated Akt also inhibits GSK3 complex formation and GTPase activity, which facilitates the mTOR signaling pathway through G protein Rheb [63,64]. This link suggests that mTOR and HIF-1α can be used as potential targets for future treatment methods of EC (Figure 2).

### 3.2. PI3K/Akt Pathway

HIF-1α is generally stabilized under hypoxic conditions, but a study on cultured skeletal muscle cells showed that HIF-1α was stabilized by insulin treatment even in normoxic conditions. Both hypoxia and insulin signaling can activate the PI3K/Akt pathway, which may stabilize HIF-1α by controlling its protein translation [65]. The PI3K/Akt pathway regulates cell growth and survival, with Akt playing a major role in regulating insulin-mediated responses like the transport of glucose, gluconeogenesis, and glycogen synthesis [66]. PTEN, a tumor suppressor gene, negatively regulates the PI3K/Akt pathway and inhibits angiogenesis.

However, it was observed that in a T2DM GK rat model, the expression level of PI3K and Akt were increased, while the expression level of PTEN was decreased in abdominal aorta, leading to the high expression levels of VEGF, which contribute to cancer progression [67]. Insulin resistance is present in almost 80% of patients with T2DM and can lead to alterations in this pathway, which influence disease prognosis [22,68]. In many of the endometrioid EC patients, this pathway is abnormally activated due to loss of PTEN [69]. Missense mutation of PTEN, a tumor suppressor gene, is the most frequently observed event in EC associated with microsatellite instability and reduces P53 overexpression [70]. Interestingly, somatic mutations in PTEN are associated with dysregulation of the PI3K/Akt pathway, leading to increased abnormal cell proliferation and function. Sensitizing cancer cells with effective inhibitors may enhance the efficacy of chemotherapy and targeted therapies, significantly impacting cancer cell viability and survival [71,72].

**Figure 3:**
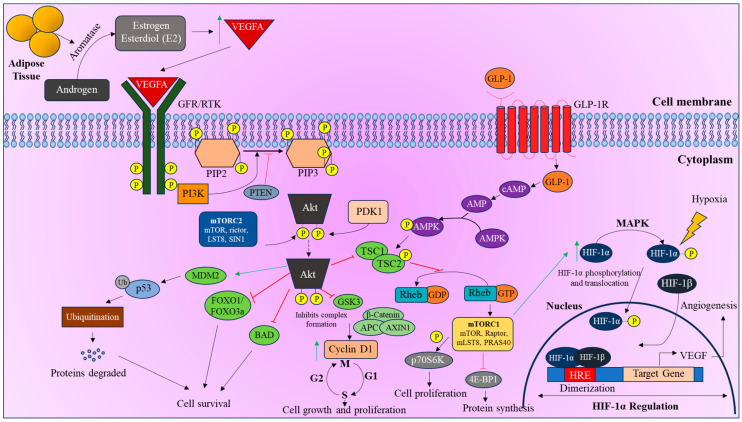
Role of PI3K/Akt/mTOR pathway in hormone-induced HIF-1α regulation. Adipose tissue releases aromatase, converting androgens to estrogen and estradiol (E2), which activates the PI3K/Akt/mTOR pathway, regulating HIF-1α by inducing VEGFA. VEGFA binds to RTK or GFR, initiating a cascade that recruits PI3K, converting PIP2 to PIP3, activating Akt. Activated Akt inhibits cell death signals, promotes cell proliferation, and supports mTOR signaling. The mTORC1 complex upregulates HIF-1α, stabilizing it even without hypoxia, leading to VEGF transcription for angiogenesis. Additionally, GLP-1 used in T2DM treatment upregulates HIF-1α via cAMP-mediated mTOR pathway induction.

Estrogen, a key factor in endometrial growth, is predominantly produced by the ovaries in pre-menopausal women, whereas the adipose tissue serves as the primary estrogen source in postmenopausal women through the conversion of androgens to estrogen and estrone by the enzyme Aromatase [73]. The increasing levels of these hormones cause an imbalance and rapidly activate the PI3K/Akt/mTOR pathway and regulate HIF-1α by inducing the VEGFA ligand. VEGFA or other growth hormones (GH) such as estrogen and leptin can bind to the receptor tyrosine kinase (RTK) or growth factor receptor (GFR) and activate a cascade to indirectly regulate HIF-1α. This cascade is initiated when RTK autophosphorylation occurs and recruits PI3K, which converts PIP2 to PIP3. Hyperinsulinemia and insulin resistance in postmenopausal women with T2DM can further elevate the levels of estrogen, which could potentially aid in the development of EC [74]. This suggests that T2DM may enhance cancer initiation and progression through abnormal sex hormone signaling [46]. Excess estrogen activates the PI3K/Akt pathway and HIF-1α, promoting the progression of cancer by inducing VEGF, a key angiogenic factor [63] (Figure 3).

MUC1, an oncoprotein having a cytoplasmic domain that interacts with the SH2 domain of PI3K, thereby plays a significant role in the transcriptional regulation of genes associated with tumorigenesis. Overexpression of MUC1 in endometrial cells, particularly in patients with diabetes, has been linked to insulin and is associated with fertility issues [75]. In EC patients, high levels of E74-like factor 3 (ELF3) have been documented, which showed a positive correlation with the MUC1/HIF-1α pathway. The interaction between ELF3 and the promoter region of MUC1 promotes EC cell proliferation, migration, and invasion. In contrast, silencing ELF3 can inhibit the migration and invasion of EC cells, suggesting its potential therapeutic target [76].

Previous research in both human and animal models has confirmed that high glucose levels lead to increased expression levels of ELF3. The upregulation of ELF3 was observed in peripheral blood mononuclear cells (PBMCs) of diabetic patients and in aortic tissue from diabetic rat models [77]. These findings suggest that targeting upstream factors in the PI3K/Akt pathway could be used as a potential target for treating ECs [78].

## 4. Regulation of HIF-1α in T2DM and EC

### 4.1. Prognosis

Comorbidity has prognostic significance in both type I and type II EC (EC), though the overall prognosis between these types. The impact of comorbidity varies with the stage of EC but remains significant regardless of type [79]. The 5-year survival rate for EC patients with DM is notably lower compared to those without DM (68% versus 84%). This effect persists even after adjusting for age, cancer stage, diagnosis period, cardiovascular conditions, and treatment. Patients with EC and DM often exhibit more unfavorable characteristics, such as advanced FIGO stage, similar recurrence rates, and poorer overall survival compared to those without DM [80].

In univariate analysis, DM and HTN were associated with reduced survival rates in EC patients. Multivariate analysis confirmed that DM and HTN remained linked to decreased survival even after adjusting for stage, age, and grade. However, factors such as body mass index, smoking habits, parity, age at menarche, and years of estrogen exposure did not impact survival either before or after adjusting for these variables [81]. Diabetes, other comorbidities, and obesity are significant adverse indicators of survival rates in women with EC [82]. For individuals diagnosed with endometrioid EC, the presence of necrosis and the expression of HIF-1α associated with necrosis are significant indicators for prognosis. Enhanced adjuvant therapy may be necessary to enhance the prognosis of patients exhibiting these characteristics [83].

Overexpression of HIF-1α is commonly observed in patients with high-grade tumors, lymphatic invasion, and myometrial invasion, and it is a strong predictor of poor prognosis in EC patients [84]. Therefore, HIF-1 could be a potential target for developing anticancer agents as it plays a key role in tumor cell adaptation to hypoxic environments by active transcription of genes that regulate processes like angiogenesis, cell proliferation, survival, metabolism of glucose, regulation of pH, and migration [85]. However, it was observed that upregulation of HIF-1α accelerated the recovery process in diabetic wounds in animal models. This effect is mainly due to HIF-1α in VEGF expression, which can enhance wound healing even in hypoxic diabetic tissues where VEGF production is impaired [86].

Another study showed that inhibiting HIF-1α in adipose tissue can alleviate obesity and insulin resistance, making HIF-1α a potential therapeutic target for obesity and T2DM. This finding was supported by studies such as metabolic efficiency, indirect calorimetry, and hyperinsulinemic-euglycemic clamp studies on high-fat diet (HFD)-induced adipocyte-specific HIF-1α knockout mice [87]. However, the role of HIF-1α in diabetic pathogenesis is complex and can vary even within the same tissues [88]. While HIF-1α targeting in EC is widely recognized and linked with improved overall survival [89], its depletion in EC patients with diabetes might impact mortality related to diabetic complications, posing an additional health challenge [90]. This complexity highlights the need for further research to unravel the complex relationship between HIF-1α and diabetes.

### 4.2. Treatment Strategies

Treatments for diabetes are independently associated with both the incidence and prognosis of EC [91]. Targeted therapies that focus on HIF-1α appear promising in EC, especially helping cancer cells adapt to low-oxygen conditions, thereby promoting tumor progression, invasion, and resistance to treatment [84]. Additionally, in patients with comorbid T2DM, elevated blood sugar levels can exacerbate the hypoxic tumor microenvironment [92]. While HIF-1α targeting could be effective in treating some cancers, it is important to consider the potential complications for diabetic patients (Table 1).

Metformin isolated from *Galega officinalis* has shown promise in cancer management [93]. It has been associated with reduced risk of cancer and lower cancer-related mortality in diabetic individuals affected by various cancer types, including EC [94]. On the other hand, metformin is known for its ability to lower glucose levels in diabetic patients and also acts on the PI3K-AKT pathway and activation of AMPK, which is associated with antitumorigenic effects [95,96]. Metformin has emerged as a potentially beneficial adjunctive treatment for EC, particularly in women with non-endometrioid tumors. Diabetic patients with EC who are treated with metformin showed a reduced risk of mortality when compared to those who are not using metformin [97].

The effects of metformin on EC risk and outcomes in diabetic patients present a mixed picture. While some studies have reported a significant reduction in the overall risk of developing EC with metformin use in a dose-dependent manner [98], other research has found no significant impact on survival outcomes for EC patients with diabetes [99]. Larger studies with a prospective randomized control design are necessary to elucidate the potential benefits of metformin in both the prevention and treatment of EC [94]. A clinical study on metformin revealed that the use of metformin has no significant impact on progression-free survival with deep myometrial and lympho-vascular invasion in EC in women with T2DM [100]. Alongside this, a dose-specific preoperative administration of metformin to patients diagnosed with EC significantly decreased the level of insulin, IGF-1, glucose, and leptin, thereby inhibiting the cancer cell progression [101]. A recent population-based analysis also suggested that metformin treatment did not improve overall or cancer-specific survival in diabetic EC patients [102]. These conflicting results indicate the need for larger, well-designed, prospective randomized controlled trials to better understand the potential benefits of metformin in the prevention and treatment of EC.

The aberrant overexpression of HIF-1α protein in numerous solid tumors makes it a significant therapeutic target [103]. Research in hypoxia-related drug therapies focuses on the development of drugs that directly target HIF-1 signaling and influence alternative signaling pathways for indirect HIF-1 regulation [104,105]. Several non-selective inhibitors that affect signaling pathways either upstream or downstream of HIF-1 have been identified. Few non-selective inhibitors like topoisomerase I inhibitor Topotecan, mTOR-inhibitor Rapamycin, and angiogenesis inhibitor Bevacizumab are known to reduce HIF-1α protein levels and improve EC management [89].

Downregulating HIF-1α could potentially exacerbate diabetic complications [88]. Therefore, for patients with both diabetes and cancer, combination therapy strategies that target HIF-1α alongside traditional cancer treatments and diabetes management might be particularly beneficial. This strategy might involve adding drugs that enhance vascular endothelial growth factor (VEGF) expression in a controlled manner alongside targeting HIF-1α regulation. Finding the right balance for combination therapy with HIF-1α inhibitors and VEGF enhancers. Upregulating VEGF could counteract the adverse effects of HIF-1α downregulation on diabetic complications, potentially leading to improved overall treatment outcomes [106].

Topotecan, a topoisomerase I inhibitor, a semisynthetic analog of camptothecin isolated from *Camptotheca acuminata* [107], prevents the translation of HIF-1α, leading to reduced protein levels and decreased transcriptional activity of HIF-1α target genes [104]. However, it is less commonly used for treating EC and is only considered when prior chemotherapy treatment has failed [108]. Topotecan has been used as a secondary treatment for ovarian cancer, being a potent small molecule inhibitor targeting HIF-1 [109,110]. It works by inhibiting both the hypoxia-induced increase in HIF-1α protein levels and its DNA binding activity [111]. A phase II clinical trial among EC patients revealed that higher doses were toxic initially, which was managed at a lower concentration of 1 mg/m^2^, leading to a median survival rate of 6.5 months [112]. Similarly, another clinical gynecological oncology study revealed that a lower dose of Topotecan has not shown a significant impact in advanced or recurrent EC in patients who previously underwent chemotherapy [108].

Rapamycin, an mTOR inhibitor from *Streptomyces hydroscopicus* AY B-994 [113], inhibits the mTOR pathway and prevents the translation of HIF-1α under hypoxic conditions [114]. mTOR itself regulates HIF-1α by facilitating the translation of HIF-1α mRNA into protein [115]. Targeting the mTOR pathway with inhibitors such as Rapamycin and derivatives like Temsirolimus and Everolimus has potential therapeutic applications in EC treatment [116]. These inhibitors of mTOR, which also target tyrosine kinases, are associated with hyperglycemia, a characteristic feature of diabetes [117]. In line with this, one of its derivatives, Temsirolimus, has been shown to have a higher therapeutic effect in chemotherapy-naive EC patients when compared to patients who had previously undergone chemotherapy treatment [118].

**Table 1:** List of compounds/drugs targeting hypoxia-inducible factor-1α.

S. No	Compound/Drug	Source	Mode of Action	Target	References
1	Metformin	*Galega officinalis*	Promotes the degradation of HIF-1α, inhibits HIF-1α accumulation by AMPK-independent activation	AMPK, GLUT4, mTOR	[88,116]
2	Topotecan	A semisynthetic analogue of camptothecin isolated from *Camptotheca acuminata*	Prevents the translation of HIF-1α; decreased transcriptional activity of HIF-1α target genes	Topoisomerase I	[96,101]
3	Rapamycin	*Streptomyces hydroscopicus* AY B-994	Inhibits the mTOR pathway and prevents the translation of HIF-1α under hypoxic conditions	mTOR	[88,117]
4	Bevacizumab	Humanized monoclonal Ab produced in Chinese hamster ovary (CHO)	Targets VEGF, a major downstream target of HIF-1α, and can reduce angiogenesis	VEGFA	[110]
5	Temsirolimus	Derivative of Rapamycin	Inhibits translation of HIF-1α	mTOR	[104,118]
6	Everolimus	Derivative of Rapamycin	Inhibits translation of HIF-1α	PI3k/AKT/mTOR	[104,118]

Bevacizumab, a humanized monoclonal antibody produced from Chinese hamster ovary (CHO) cells [119] targeting VEGF, is a major downstream target of HIF-1α and can reduce angiogenesis and tumor growth [120]. Phase II clinical trials have shown promising results for Bevacizumab in treating recurrent or persistent EC, showing a progression-free survival for 6 months in comparison with patients who underwent prior radiation [121,122,123]. High levels of HIFs are associated with resistance to conventional cancer therapies, such as chemotherapy, radiation therapy, and disease progression [89].

Hence, combining HIF inhibitors with current therapies could potentially increase anti-tumor activity and reduce treatment resistance, as observed in many cancers like brain, lung, colorectal, liver, and breast cancer [124]. However, it is important to note that direct HIF-1α inhibitors are still in development, and further research is needed to optimize these treatment strategies [89,125].

## 5. Conclusions

HIF-1α, a key regulator of cellular response to hypoxia, is emerging as a promising biomarker in EC, especially in patients with T2DM implicating both diseases, suggesting a potential molecular relationship that warrants further investigation. Although agents targeting the HIF-1 pathway, such as Bevacizumab and mTOR inhibitors, show promise, directly targeting HIF-1α may increase diabetes complications. For therapies targeting HIF-1α to be effective, patients with EC and diabetes should be tested for prognostic markers such as HIF-1α expression levels. Targeting HIF-1α could potentially mitigate diabetic complications associated with mortality in these patients, but careful management and patient selection are important considerations. Alongside this, targeting HIF-1α through inhibitors may increase the anti-tumor effect, thereby reducing the resistance to conventional cancer treatment strategies and halting the progression. Further research is needed to develop optimal treatment strategies, including exploring direct HIF-1α inhibitors, evaluating the impact of metformin in comorbid conditions, and refining combination therapies to minimal side effects. Ultimately, effective targeting of HIF-1α, along with other potential biomarkers, may enhance patient outcomes, reduce mortality, and alleviate the socioeconomic burden of endometrial cancer (EC) in individuals with type 2 diabetes mellitus (T2DM), paving the way for more precise treatment strategies.

## Abbreviations

The following abbreviations are used in this manuscript:

cAMP	Cyclic adenosine monophosphate
CBP	CREB-binding protein
EC	Endometrial cancer
GLP1	Glucagon-like peptidide 1
HIF	Hypoxia-inducible factor
HRE	Hypoxia-responsive element
MAPK	Mitogen-activated protein kinase
mTOR	Mechanistic target of Rapamycin
OS	Oxidative stress
OXPHOS	Oxidative phosphorylation
PDK1	3-phosphoinositide-dependent kinase 1
PI3K	Phosphoinoside 3-kinase
PTEN	Phosphate and tensin homolog
RHEB	Ras homolog enriched in brain
RTK	Receptor tyrosine kinase
VEGF	Vascular endothelial growth factor
